# Effects of workload on 3D joint moments in cycling and their implications for injury prevention

**DOI:** 10.3389/fbioe.2025.1657558

**Published:** 2025-09-12

**Authors:** Ezequiel Martín-Sosa, Juana Mayo, Joaquín Ojeda

**Affiliations:** ^1^ Departamento de Ingeniería Minera, Mecánica, Energética y de la Construcción, Escuela Técnica Superior de Ingeniería, Universidad de Huelva, Huelva, Spain; ^2^ Departamento de Ingeniería Mecánica y Fabricación, Escuela Técnica Superior de Ingeniería, Universidad de Sevilla, Sevilla, Spain

**Keywords:** cycling, statistical parametric mapping, 3D joint moment, 3D joint angle, power effect, statistical analysis, injury prevention

## Abstract

**Background and Introduction:**

Several studies have been published in the literature analysing the effect of workload on joint moments during pedalling. Most of these studies focus on a single plane, mainly the sagittal plane, or on one joint (the knee). In this work a workload effect analysis is proposed on the three main joints of the lower body and on the three anatomical planes. Usually, this type of study has been carried out using 0D metrics such as the maximum or the variable range. In this work the analysis has been extended by complementing it with a statistical parametric mapping analysis.

**Methodology:**

Ten participants pedalling at 90 rpm and three pedal powers (170, 240 and 310 W) were analysed.

**Results:**

The results obtained show that pedalling power affects the moments in the three joints and in the three anatomical planes.

**Discussion:**

Analysis of the common causes of the main overload injuries that occur in cycling suggests that a three-dimensional analysis of the joint moments of the three main joints of the lower limb, including 0D and 1D data, is useful for the prevention of these injuries.

## 1 Introduction

Cycling is a very popular sport with multiple advantages for human health. Apart from recreational or sporting uses, one of the most important applications of cycling is its use as a treatment in rehabilitation processes. This practice is justified in the literature because the main loads on the lower body occur in the sagittal plane and these can be much smaller than those produced in other activities such as walking (Kutzner et al., 2012; Ericson et al., 1986).

However, studies such as that carried out by Ericson showed that knee loads outside the sagittal plane, although lower, can be of the same order as those produced during walking (Ericson et al., 1984). Additionally, there are many occasions in which users tend to pedal under inappropriate conditions: non-optimal stems, incorrect saddle height or position of the foot on the pedal, etc. All these factors can lead to injuries, loss of performance or relapses (Thorsen et al., 2020; Bini et al., 2010a). The most common injuries caused by the aforementioned factors are iliotibial band syndrome, hip trochanteric bursitis, ilipsoas tendinitis, knee patellofemoral syndrome, Achilles tendonitis or foot metatarsalgia (Thompson and Rivara, 2001). Therefore, an accurate understanding of the effect of pedalling at different powers on lower body joint moments can help to prevent lower limb joint overload injuries as well as to optimise the return to sporting activity after locomotor pathologies within the limits of joint safety. The works found in the literature that analyse the pedalling power effect on lower body joint moments are those by Ericson et al. (1986), Mornieux et al. (2007) and Fang et al. (2016). In the literature there are also works that analyse the effect of pedal power and cadence in conjunction on kinematics or muscle forces (Bini et al., 2010a; Bing et al., 2024; Pouliquen et al., 2018; Pouliquen et al., 2021), or the effect of saddle height (Bini et al., 2010a; Bing et al., 2024; Bini, 2021), the fatigue onset (Sanderson and Black, 2003; Bini and Diefenthaeler, 2010; Bini et al., 2010b; Galindo-Martínez et al., 2021; Bartaguiz et al., 2023; Holliday et al., 2023) or crank length (Barratt et al., 2016; Park et al., 2022).

Ericson et al. (1986) set out to analyse the effect of changing workload, cadence and saddle height on hip and knee moments. To do so, they analysed 6 voluntary participants at a recreational level. The results analysed in this work are the maximum and minimum values of the moments at different pedalling powers for the sagittal plane moments of the hip and knee. The authors concluded that the modification of the pedalling power is the most influential factor in the variation of the maximum value of the hip and knee flexion moment.

The work carried out by Mornieux et al. (2007) aims to determine the robustness of the relative distribution of the joints of the lower body in the net joint moments, analysing 7 volunteer participants. As results, the relative distribution of the joints in the net joint moment and the temporal evolution of the flexion-extension joint moments for different power and pedalling cadences were obtained. In this work authors concluded that the change of workload and/or cadence hardly affects the relative distribution of the joint moments but it does affect the flexion-extension moments in the joints.

There are few studies in the literature that analyse the effect of power outside the sagittal plane. Fang et al. (2016) studied, for rehabilitation purposes, the effect of power and cadence in the sagittal and frontal plane of the knee. For this purpose, 18 volunteer participants were evaluated. The findings of this study indicated that increased workload at a constant cadence resulted in greater peak knee abduction moment, peak knee extension moment, and knee abduction range when increasing the workload from 0.5 to 2.5 N. Another interesting finding was that the participants in this study demonstrated two different frontal plane knee moment patterns. Some participants presented an abduction moment, while the others exhibited an adduction moment throughout the power phase.

Once the most recent works found in the literature that study the effect of pedalling power on the joint moments of the lower body have been analysed, it can be affirmed that information on the effect of power has been found for the hip, knee and ankle joint moments in the sagittal plane. Additionally, for the knee, this information is extended to the frontal plane. This may be because most driving power and force comes from knee extension during the push phase and flexion during the recovery phase (Faria et al., 2005). However, pedalling at higher powers causes an increase in hip flexion and ankle plantarflexion joint moments (Bini and Diefenthaeler, 2010; Bini et al., 2010b), which may affect joint moments in other anatomical planes because the lower body acts as a closed kinematic chain during pedalling (Galindo-Martínez et al., 2021).

Another conclusion reached is that previous works analyse the effect of power on joint moments carried out a statistical study using 0D variables (ranges of moments, maximum and minimum values and cycle average). This methodology is widely accepted by the scientific community (Bini et al., 2010a; Mornieux et al., 2007; Fang et al., 2016; Bini and Diefenthaeler, 2010; Bini et al., 2010b; Faria et al., 2005). However, this approach may miss important information if the variable analysed is of a continuous type. To avoid this, one-dimensional Statistical Parametric Mapping (SPM) which can evaluate entire movement is employed. SPM has been widely used in neuroimaging, since it allows to see changes in the whole brain, not just in specific areas (Penny et al., 2007). This approach has advantages over statistical analysis using 0D data, which may miss generalised changes.

In the field of biomechanical analysis of cycling, the number of studies using SPM to carry out statistical analyses is increasing (Bing et al., 2024; Pouliquen et al., 2021; Bini, 2021; Galindo-Martínez et al., 2021; Park et al., 2022). The first of these works was by Bini (2021) in which the effect of varying the saddle height on the movements and joint moments and forces contained in the sagittal and transverse planes of the knee is analysed. Another outstanding work using the SPM is by Galindo-Martínez et al. (2021), in which the effect of the onset of fatigue on the 3D kinematics of the lower body and trunk of the participants is analysed. Park et al. (2022) in their work apply SPM to both joint angles and moments in the sagittal plane as well as muscle forces, in order to analyse the effect of crank length, but in standing cycling. Pouliquen et al. (2021) and Bing et al. (2024) use SPM to analyse the effect of fatigue and the effect of workload and saddle height on muscle forces respectively. In all these works (Bing et al., 2024; Pouliquen et al., 2021; Bini, 2021; Galindo-Martínez et al., 2021; Park et al., 2022) the analysis using SPM allows the authors to identify the intervals in which the study variables are affected by the analysed factors.

All the above mentioned motivates the main objective of this work, the analysis of the effect of power on the hip, knee and ankle joint moments, in the 3 anatomical planes, frontal, transverse and sagittal. As a secondary objective, the results obtained will be used to obtain information to help prevent overuse injuries. In order to carry out this study, a statistical analysis will be performed using traditional methodology (0D data) and also SPM methodology. The starting hypotheses for this study are:

• The workload effect on joint moments using the traditional methodology will be different to the power effect on the same joint moments using SPM methodology.
• Pedalling at different workloads will influence the 3 joints that compose the lower body.
• Pedalling power will affect the 3 anatomical planes.

## 2 Materials and methodology

### 2.1 Participants

In order to achieve the objectives established in this study, 10 participants were analysed, all men, adults, with a mean age of 26.87 ± 4.97 years, a mean height of 1.74 ± 0.07 m, a mean weight of 67.89 ± 11.34 kg and a mean BMI of 22.33 ± 3.18 kg/m2. The participants were volunteers and reported that they used the bicycle as a means of transport, without seeking to improve their sport or performance. The sample size was based on previous studies found in the literature (Mornieux et al., 2007; Pouliquen et al., 2018; Carpes et al., 2007). The inclusion-exclusion criteria were as follows:

Inclusion criteria:

• Participants aged 18 years or older.
• Leg length discrepancy (dysmetria) less than or equal to 5 mm.
• Body size compatible with the test bicycle.
• Intrinsic Q-factor similar to the standard Q-factor of the bicycle.

Exclusion criteria:

• Diagnosed locomotor or cardiopulmonary conditions that could interfere with test performance.
• High-performance or professional cyclists.

To assess the discrepancy between lower limbs, each lower limb was measured using the methodology developed by Davis et al. (1991) which is used by other authors in the cycling biomechanics analysis (Bini et al., 2016). On the bicycle, the Q-factor is defined as the distance between the force application points on the pedals. In general, the standard Q-factor used by bicycle manufacturers is 250 mm. In participants, this factor is determined from the distance between the two anterior superior iliac spines (Millour et al., 2021). The participants signed a consent form approved by the Andalusian Biomedical Research Ethics Platform (code number 0230-N-22).

### 2.2 Experimental data post-processing

Experimental data were collected and processed with Vicon Nexus^®^ 2.12.1 commercial software. Based on residual analysis (Winter, 2009), the data were filtered with a low-pass filter (6 Hz, 4th order Butterworth filter) by a custom-made routine using Matlab R2024a (The MathWorks, Inc., Natick, MA, United States) to eliminate high-frequency noise (Fang et al., 2016; Gregersen and Hull, 2003).

**FIGURE 1:**
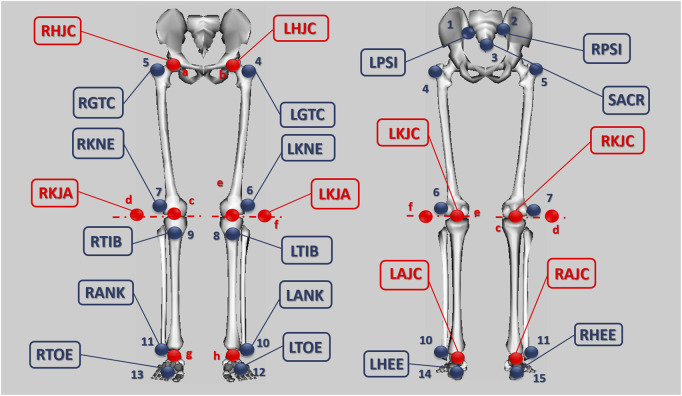
Location of markers that form the protocol. Blue: physical markers. Red: virtual markers.

In order to obtain the joint moments, the inverse dynamic problem had to be solved, so the lower body movement kinematics and the reaction forces with the pedals were previously obtained. The kinematic analysis was carried out using the marker protocol developed by Martin-Sosa et al. (2025) (Figure 1). This protocol focuses on the lower body and models it using 7 solids: pelvis, right and left thigh, right and left shank and right and left foot. As shown in Figure 1, in the pelvis two markers were placed on the posterosuperior iliac spines (RPSI and LPSI) and one marker on the sacrum (SACR). The thighs had one marker placed at the greater trochanter (RGTC and LGTC), one at the lateral epicondyle (RKNE and LKNE) and one virtual marker at the hip joint centre (RHJC and LHJC). The legs had one marker placed on the tibial tuberosity (RTIB and LTIB), one on the lateral malleolus (RANK and LANK) and defining the axis of rotation of the knee joint two virtual markers were used per side (RKJC-RKJA and LKJC-LKJA). In the foot the following markers were placed: one on the calcaneus (RHEE and LHEE), one on the second phalanx of the first metatarsal (RTOE and LTOE) and finally one defining the ankle joint centre (RAJC and LAJC). In total 15 physical markers and 8 virtual markers were used. This protocol did not impose kinematic constraints.

The forces on the pedals were recorded from the measuring equipment developed by Martín-Sosa et al. (2021) in their work. This equipment had a measurement error of less than 4%, similar to those used and developed by other works (Balbinot et al., 2014). Measurement equipment was installed on each pedal. The inverse dynamic problem was solved applying the Newton-Euler equations and using the bottom-up procedure (Winter, 2009). The joint moments were expressed in the local system of the joint parent solid, more proximal solid.

### 2.3 Instrumentation

**FIGURE 2:**
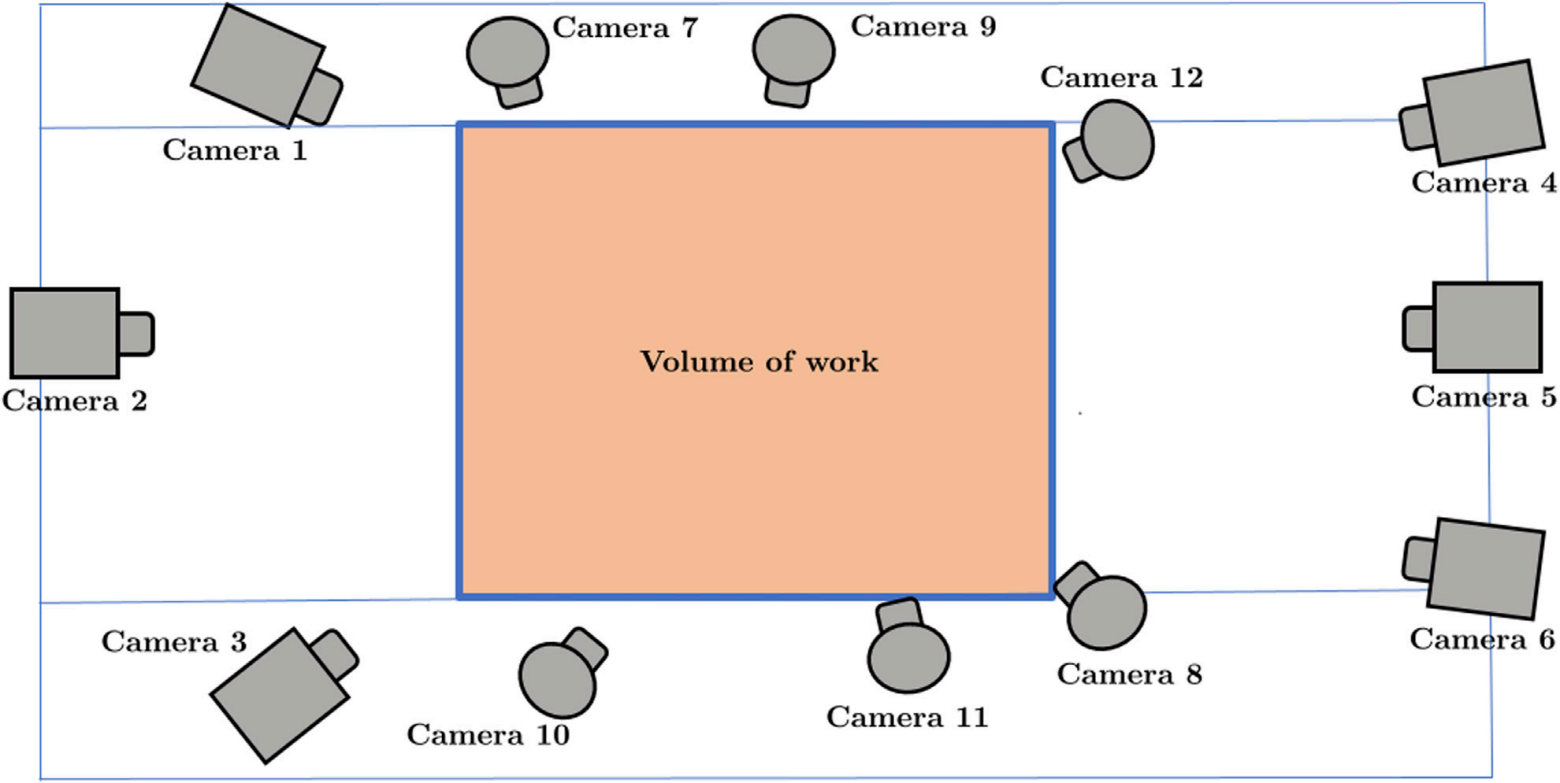
Camera layout in the laboratory.

**FIGURE 3:**
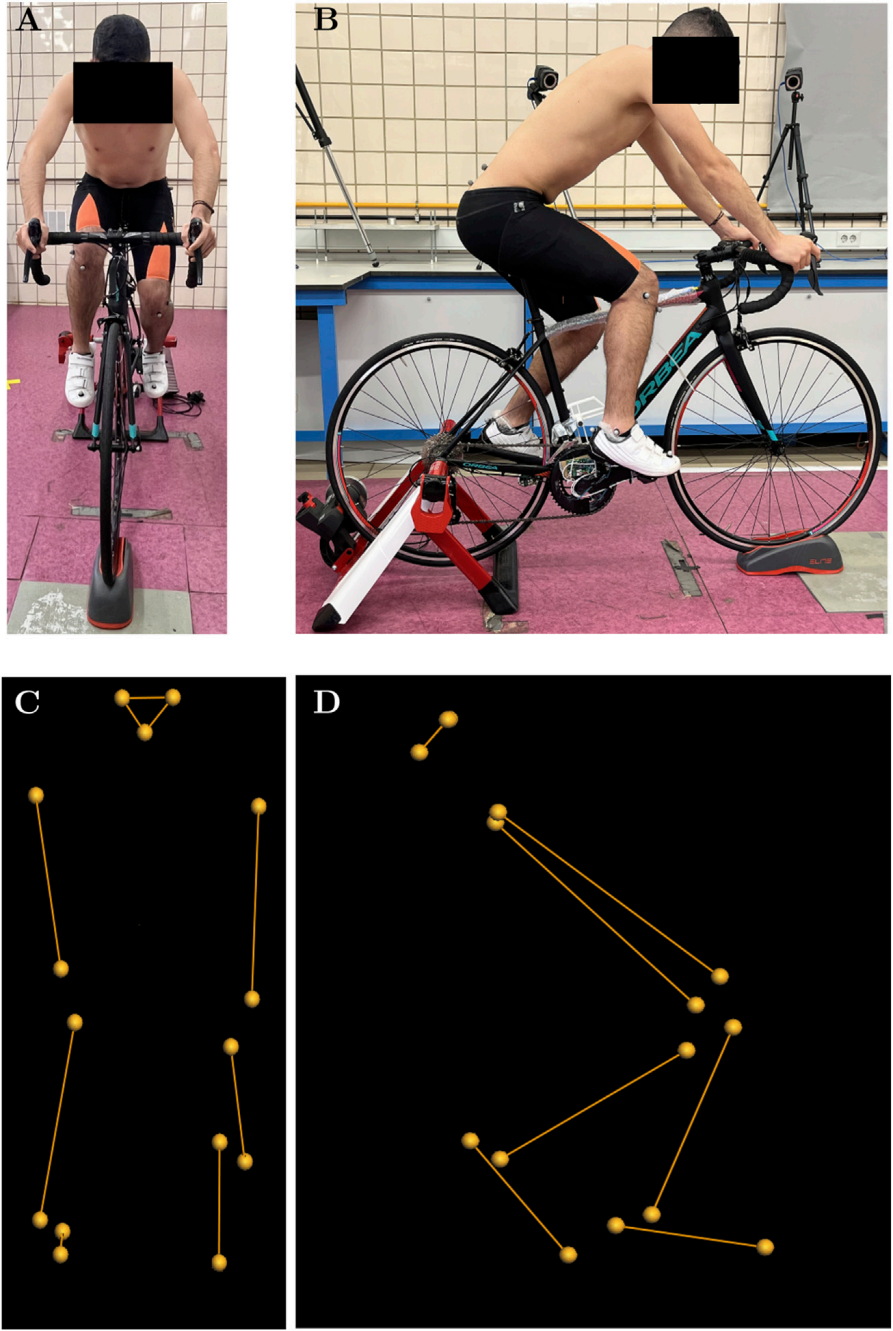
Detail of the participant during the tests and the reconstruction performed in Vicon Nexus^®^. **(A)** Frontal view of the participant during the tests. **(B)** Sagittal view of the participant during the tests. **(C)** Reconstruction of the physical markers placed on the participant seen in frontal view. **(D)** Reconstruction of the 19 physical markers placed on the participant seen in the sagittal plane.

A motion capture system comprising twelve infrared cameras (100 Hz; Vicon Motion Systems Ltd., Oxford, United Kingdom) and retroreflective markers of 14 mm diameter was employed. Figure 2 shows a layout of the position of the cameras, their relative position to each other, and the working volume. Six of the twelve cameras were MXT010 models, which are high-resolution digital units capable of sampling at up to 250 Hz. Owing to their larger physical dimensions, these cameras were typically mounted at elevated positions—approximately 3 m above ground level—and at a distance of 2.5–3 m from the calibrated capture volume, cameras 1–6 in Figure 2. The remaining six units were Bonita models, also high-resolution digital cameras, though with a maximum sampling rate of 100 Hz. Their more compact form factor permitted placement on tripods at ground level, approximately 1.5 m from the participant, cameras 7–12 in Figure 2. Given the discrepancy in maximum sampling frequencies between the two camera models, the overall system sampling rate was standardised to 100 Hz during calibration. All twelve cameras were synchronised via a central control unit (Giganet, Vicon^®^), which also served to transmit recorded data to the acquisition computer. Subsequent data processing was carried out using Nexus software version 2.12.1 (Vicon Motion Systems Ltd., Oxford, United Kingdom). Figures 3A,B shows the participant during the tests. Figures 3C,D reflects the marker cloud obtained after processing the data in the Vicon Nexus^®^ software.

The experimental procedures were conducted using a commercially available Orbea H50 bicycle, which was mounted on a stationary training roller (Elite Novo Force; Elite S.R.L., Fontaniva (PD), Italy). For the purposes of testing, the bicycle was fitted with clipless pedals, and participants wore cycling shoes equipped with compatible cleats. These shoes were provided to all participants. Cleat positioning was verified individually for each participant in accordance with the method proposed by Bini and Carpes (2014), which defines the optimal position as aligning the pedal axle with the plantar fat pads. To confirm this alignment, a preliminary assessment was carried out prior to motion capture. During this pre-test, participants cycled at a low cadence under high resistance to facilitate identification of the foot region exerting the greatest pressure on the pedal. If this region coincided with the recommended position, no modifications were made. Otherwise, cleat position and orientation were adjusted accordingly, and the procedure was repeated. All assessments and adjustments were conducted by the same researcher for all ten participants in order to minimise inter-rater variability related to cleat alignment. The bicycle model, shoe model, and training roller model can be seen in Figures 3A,B.

### 2.4 Test conditions

The pedalling conditions (power and cadence) were chosen according to the participants physical condition to avoid the fatigue appearance shortly after the test started. The cadence was set at approximately 90 rpm and controlled by acoustic signals. Three pedalling powers were chosen: 170 W (P1), 240 W (P2) and 310 W (P3), which were modified using the friction of the roller on the rear wheel.

In addition, a pulse oximeter was employed alongside individual assessments of maximal heart rate. Participants also completed a basic health questionnaire, which included questions regarding their personal history of injury. Cardiopulmonary auscultation was conducted, and both oxygen saturation and resting heart rate were measured. Furthermore, the Medical Research Council (MRC) dyspnoea scale during physical exertion was administered, as it is considered appropriate for populations comprising non-athletes.

In this work, saddle height was established using the methodology developed by Holmes et al. (1994), which was based on the measurement of knee flexion in static measurements. However, as dynamic measurements were taken, this methodology was modified following the recommendations of Ferrer-Roca et al. (2012), Ferrer-Roca et al. (2014) to adapt it to dynamic measurements. To achieve greater comfort for each participant, the saddle height could be altered slightly, trying, in most cases, to ensure that the minimum angle of knee flexion was within the range established (Holmes et al., 1994; Ferrer-Roca et al., 2012; Ferrer-Roca et al., 2014). Those participants who, after this slight modification, had a minimum knee flexion higher or lower than that reported in the literature (Holmes et al., 1994; Ferrer-Roca et al., 2012; Ferrer-Roca et al., 2014) were excluded from the study. This behaviour indicated that the size of the bicycle was not suitable for these participants, which is why this fact is included in the inclusion-exclusion criteria. A single saddle model was used for all participants throughout the study. This was the standard saddle supplied with the bicycle, measuring 16 cm at its widest point and 5 cm at its narrowest. The saddle featured an anatomical cut-out to relieve perineal pressure. The decision to standardise the saddle across all participants is supported by previous research on bicycle fitting (Kotler et al., 2016; Cyr and Ascher, 2023), which highlights the relevance of saddle design, particularly during extended cycling sessions. However, in the present study, cycling duration was limited to 10–15 min, thereby reducing the likelihood of saddle-related discomfort. Nonetheless, participant comfort was assessed through verbal feedback before, during, and after testing, and no reports of discomfort attributable to the saddle were recorded.

The effect of power on participants was analysed by having each participant perform three tests, one for each selected power level, starting with the lowest power level (P1) and ending with the test requiring the highest pedalling power (P3). All these tests were carried out on the same day, and in order to avoid fatigue, the time between tests ranged from 5 to 10 min. Each test was divided into three parts. The first part, covering the first 2–3 min of each test, was used as a warm-up to adapt to the new pedalling conditions and to verify that the participant felt comfortable pedalling with the selected height and position of the saddle and cleats on the bike. In this first part, the cadence was set at 60 rpm, while the power was set at 75 W below the target power for each test. Once the participant had acclimatised to the new conditions, the second part of the test began. This second part started with a gradual increase in cadence and power. The cadence increased linearly for 90 s until it reached a cadence of 90 rpm. Once the participant reached a stable cadence, the pedalling power was modified, which increased for 60 s by modifying the resistance of the roller. Once the desired pedalling power was achieved, a reasonable amount of time (60–75 s) was allowed for the participant to acclimatise to the conditions. Once stabilised, the third part of the test began. Only in this part were recordings of the pedalling movement made, as the aim was to analyse the joint moments under stable cadence and power conditions. To prevent fatigue from affecting the results obtained, only five captures were recorded for each resistance, each lasting 10 s for each participant. The time between recordings was approximately 10–15 s. Both the duration of the recordings and the time between recordings were determined using a stopwatch and Vicon Nexus^®^ software. In total, for each test, participants pedalled at the target cadence and power for around 5 min. This was sufficient time to make the necessary recordings. Once the captures were made, the participant slowly reduced the cadence until reaching 60 rpm again, at which point the pedalling power was reduced to 90 W. The participant maintained these pedalling conditions for approximately 90–120 s, after which time the participant was free to reduce the cadence at will until finally stopping completely. Once the participant dismounted from the bicycle, the test was considered complete.

### 2.5 Statistical analysis

Previous to the analysis of the results obtained, the elimination of outliers was carried out through the method known as box and moustache. The analysis of the results was performed by defining one factor, pedalling power, with three levels (P1, P2 and P3), both for the statistical analysis using SPM methodology and 0D variables. For each level, the variables analysed were the temporal evolution of the joint moments in the three anatomical planes of the three joints forming the right leg, for the 1D study. For the 0D study, the variables analysed are the range and the maximum value of the same joint moments as in the previous case.

To study the effect of power on the 1D variables analysed, a statistical parametric mapping analysis was carried out using the open source spm1d statistical package (http://www.spm1d.org), in Matlab^®^ R2023a (The MathWorks, Inc., Natick, MA, United States). The SPSS^®^ software was used to study the effect of the powers on the 0D data.

A similar approach was used for both types of variables. In a first step, the Shapiro-Wilk test was applied, and the normality hypothesis was fulfilled for all cases. Subsequently, homogeneity was analysed, which was only fulfilled for some variables. To determine whether pedalling power had an effect on the variables defined in this study, a comparison of means was initially carried out using a one-factor ANOVA analysis. Components that did not satisfy the homogeneity hypothesis were subjected to Welch’s test. In all studies the significance level was set at a value α = 0.05. For those variables where significant results were obtained, a *post hoc* Bonferroni’s paired-samples was considered. For cases of non-homogeneity, the Games-Howell *post hoc* test was applied. Finally, the effect size would be calculated using Cohen’s d (Cohen, 1988) defining a large effect size for a d value >0.8 (Bini, 2021).

## 3 Results

### 3.1 Statistical analysis based on 0D data

Table 1 shows the average value and standard deviation of the range, maximum (Max) and minimum (Min) values of the joint moments contained in the three anatomical planes of the hip, knee and ankle for the different pedalling powers.

**TABLE 1:** Mean and standard deviation of the range, maximum and minimum value of the joint moments for the 3 powers output and *p-value* for the different statistical analysis.

Variable			P1	P2	P3	*P*
HIP	FL	Range	51.89 ± 8.45	77.87 ± 21.09	106.19 ± 21.83	**<0.001** ^a^ ^,^ ^b^ ^,^ ^c^
		Max	−8.45 ± 17.49	−3.32 ± 16.99	−4.80 ± 8.16	0.793
		Min	−60.35 ± 23.40	−81.19 ± 30.30	−110.98 ± 25.65	**<0.001** ^b^
	AD	Range	13.98 ± 3.38	26.41 ± 8.07	37.91 ± 7.72	**<0.001** ^a^ ^,^ ^b^ ^,^ ^c^
		Max	3.58 ± 5.45	5.33 ± 5.96	9.19 ± 9.85	0.235
		Min	−10.40 ± 5.92	−21.09 ± 10.88	−28.72 ± 12.01	**0.001** ^b^
	INT	Range	7.61 ± 2.75	13.65 ± 6.72	19.67 ± 11.10	**0.005** ^b^
		Max	1.84 ± 2.80	2.14 ± 2.70	2.52 ± 2.28	0.842
		Min	−5.77 ± 4.10	−11.51 ± 8.61	−17.15 ± 12.25	**0.024** ^b^
KNEE	FL	Range	63.26 ± 11.99	92.98 ± 17.90	112.45 ± 15.69	**<0.001** ^a^ ^,^ ^b^ ^,^ ^c^
		Max	30.84 ± 13.86	41.91 ± 16.37	53.90 ± 15.31	**0.008** ^b^
		Min	−32.43 ± 17.57	−51.07 ± 21.18	−58.55 ± 15.94	**0.011** ^b^
	AD	Range	14.97 ± 6.21	19.45 ± 3.87	26.78 ± 5.40	**<0.001** ^b^ ^,^ ^c^
		Max	2.59 ± 1.52	2.87 ± 1.63	2.84 ± 2.21	0.929
		Min	−12.38 ± 6.40	−16.58 ± 4.13	−23.94 ± 5.71	**<0.001** ^b^ ^,^ ^c^
	INT	Range	10.56 ± 4.93	14.24 ± 7.16	20.51 ± 10.14	**0.025** ^b^
		Max	9.81 ± 6.17	12.75 ± 6.03	18.06 ± 7.77	**0.033** ^b^
		Min	−0.75 ± 3.47	−1.50 ± 2.83	−2.44 ± 4.34	0.646
ANKLE	DFL	Range	17.20 ± 3.76	25.48 ± 8.82	37.42 ± 12.16	**<0.001** ^a^ ^,^ ^b^
		Max	−3.96 ± 3.36	−3.29 ± 1.87	−2.58 ± 2.44	0.509
		Min	−21.16 ± 3.63	−28.76 ± 8.88	−40.00 ± 12.06	**<0.001** ^b^ ^,^ ^c^
	INV	Range	9.69 ± 3.37	16.41 ± 6.14	23.77 ± 7.13	**<0.001** ^a^ ^,^ ^b^ ^,^ ^c^
		Max	−4.66 ± 2.26	−4.40 ± 1.72	−3.52 ± 1.23	0.275
		Min	−14.36 ± 4.49	−20.81 ± 7.50	−27.29 ± 7.91	**<0.001** ^b^
	INT	Range	6.26 ± 1.53	8.35 ± 2.08	9.57 ± 2.62	**0.006** ^b^
		Max	2.22 ± 1.44	3.87 ± 2.00	4.58 ± 2.47	**0.042** ^b^
		Min	−4.04 ± 1.73	−4.48 ± 1.85	−4.99 ± 2.25	0.561

^a^ Significant differences between P1 and P2.

^b^ Significant changes from P1 to P3.

^c^ Significant changes between P2 and P3. Abbreviation: AD: Adduction. DFL: Dorsiflexion. FL: Flexion. INT: Internal Rotation. INV: Ankle Inversion.Bold values indicate statistical significance.

Table 1 also shows the p-value of the analysis of comparison of means (ANOVA or Welch’s test). The table also shows the results of the *post hoc* analysis in the pairwise comparison. Significance is obtained for the ranges for all the joints and anatomical planes. The *post hoc* analysis shows significance for all the comparisons in case of hip flexion and abduction, knee flexion and ankle inversion. Concerning the minimum values, there is less significance than in the case of the range. For these variables the analysis of variance showed significance for all variables except for knee internal rotation and ankle internal rotation. The effect of workload on maximum values of the joint moments is smaller than for the minimum values. In this case, significance was only obtained in knee flexion and internal rotation and ankle internal rotation.

### 3.2 Statistical parametric mapping

**FIGURE 4:**
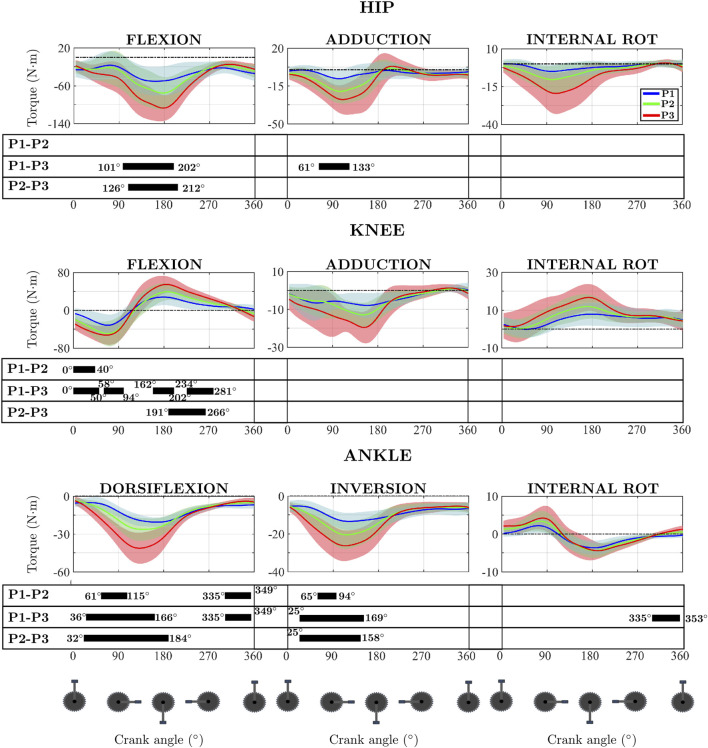
Mean and standard deviation of the hip, knee and ankle joint moment of flexion, adduction and internal rotation for the 3 powers output. Results are presented from top dead centre to bottom dead centre. The pedal cycle intervals where significant differences appeared are presented below each graph in the form of a solid black bar.

Figure 4 shows the significant results (p < 0.05) of the SPM repeated measures ANOVA comparing the 3 powers analysed. Significant results were obtained for all three anatomical planes. In the sagittal plane there are significant differences for hip, knee and ankle. In the frontal plane, differences were only obtained for the hip abduction moment and the ankle inversion moment. The transverse plane shows significant results only for ankle.

The knee flexion moment and the ankle dorsiflexion and inversion moments show significant differences for the three comparisons. The hip flexion moment shows significance in only 2 comparisons (P1–P3 and P2–P3). The hip adduction moment shows significant results for the comparison between P1–P3. The moment in the transverse plane of the ankle only shows significance for one comparison, P1–P3.

Performing the paired study using the SPM methodology made possible the identification of the intervals in the pedalling cycle at which significant differences occurred (Figure 4). In the hip flexion moment, the intervals tend to be close to the bottom dead centre (BDC). In the P1–P2 comparison, the statistically significant interval ranged from 101° to 202° of crank angle, covering approximately 100°, which represents around 30% of the pedalling cycle. A similar pattern was observed in the P1–P3 comparison, where significance was found between 126° and 212°, spanning nearly 90°, or about 25% of the cycle. Regarding the knee flexion moment, for the comparison between P1-P2 and P1-P3, it shows a greater number of intervals in which there are significant differences, although these intervals are also close to the top dead centre (TDC) and 90° (maximum tangential force). Of these two comparisons, only P1-P3 shows significant results near the BDC. For the comparison between P2 and P3, significance was only found in the recovery phase. SPM provides significant results in all three anatomical planes. In the sagittal and frontal planes, significant differences are found in during the push phase. Additionally, significant results are obtained at the end of the recovery phase in the sagittal plane and in the coronal plane. All the intervals shown in Figure 4 showed a Cohen’s D greater than 0.8.

## 4 Discussion

The main objective of this work was to analyse the pedalling power effect on the joint moments of the hip, knee and ankle joints in the 3 anatomical planes using a statistical analysis with 0D variables and a parametric statistical analysis, comparing the results obtained by each method.

### 4.1 Hip joint moments

The analysis of the 0D results in hip flexion moment is of interest when focusing on the minimum values (hip extension) because these occur during the push phase of the cycle. The results obtained in the pairwise comparisons indicate that the differences obtained in the minimums can only be explained by the change in pedal power when comparing P1–P3. This result suggests that when reaching a certain level of workload, the intravariability of the participants increased significantly, probably due to the lack of technique. Significant differences are also obtained in peak hip abductor moment, peak hip external rotation moment and in the range of the three hip joint moments when comparing P1–P3. The range for hip moments, in the P1-P3 comparison, was increased by 54.30 Nm (105%) for the flexion moment, by 23.93 Nm (171%) for the adduction moment, and by 158% (12.06 Nm) for the internal rotation moment.

The SPM results for the hip joint only showed significant results for the flexion-extension moment around the BDC (P1–P3 and P2–P3). The significance obtained in the push phase is consistent with the results obtained from the 0D analysis relative to the extension moment maximum.

The SPM give significant results outside the sagittal plane in the frontal plane but not in the transversal plane. These results can be explained by analysing the temporal evolution of the adductor and internal rotation moments. Focusing on the analysis of the maximum abductor moment and the maximum external rotation moment, the instants at which these peaks occur shift in time as workload increases. In both cases the maximum shifts to the right (about 15° between P1 and P3) as power increases. This effect is neglected when a statistical analysis based on 0D variables is carried out, which does not consider the instant where the peaks occur. In this way, the influence of workload on the temporal evolution pattern of the joint moments is not considered. According to the SPM results, changes in the temporal evolution of hip abduction moment can be explained by variations in pedalling power but these changes cannot be explained in case of the external rotation moment. The reason is the higher level of intravariability that can be observed in the internal rotation moment.

**FIGURE 5:**
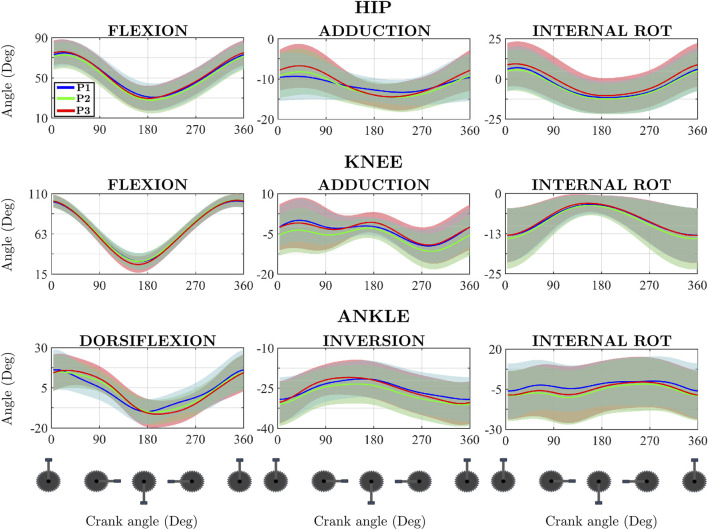
Mean and standard deviation of the temporal evolution of the hip, knee and ankle joint angle for the sagittal, frontal and transverse plane during a pedalling cycle for the three pedal powers analysed. Results are presented from top dead centres to bottom dead centre.

The analysis of the effect of power on joint moments is of interest in the prevention of overuse injuries. In the case of the hip, the most common complaints are hip trochanteric bursitis, which is promoted by repetitive hip movements. Increasing the level of load increases the risk of this ailment (Van der Plas, 1986). Iliotibial tract band friction syndrome is also very common, caused by the friction of this with the lateral femoral condyle causing irritation. This typically occurs when the knee is within 30° of full extension (Figure 5) (Farrell et al., 2003) which coincides with the moments of maximum hip and knee extension and is due to the repetition of the flexion-extension movement in these joints (Figure 4) (Wanich et al., 2007). In light of the results obtained, it can be observed that in the region of maximum knee extension, there exists a quasi-linear relationship between the increase in pedalling power and the increase in peak hip extension moment, with both variables increasing in approximately the same proportion. A similarly linear relationship is evident between the increase in pedalling power and the increase in peak knee flexion moment, although the increase in knee flexion moment is slightly lower than the increase in pedalling power (Figure 4; Table 1). Accordingly, these findings support the development of practical pedalling power adjustment strategies to prevent iliotibial tract band friction syndrome. For instance, limiting pedalling power during the push phase by adjusting gear ratios or increasing cadence to reduce joint loading or adjusting saddle height to limit knee extension may help mitigate the risk.

Outside the sagittal plane, hip abductor weakness has been linked to the development of injuries (ITB friction syndrome, anterior cruciate ligament injury) (Kotler et al., 2016). The hip abductors act to stabilise the pelvis and prevent knee joint overload. In the coronal plane, hip abductor weakness can lead to increased movement, decreasing power and increasing the injury risk of the cyclist (Kotler et al., 2016). The combined use of both 0D and 1D data methodologies can provide information on increased peak moments or alterations in the force pattern due to, for example, abductor weakness. An analysis of the obtained results indicates that an increase in pedalling power is associated with an increase in hip abduction moment (Table 1). Additionally, a shift in the moment pattern is observed, as the peak abduction moment tends to occur later in the pedalling cycle as power increases (Figure 4). One of the main advantages of using SPM in this study was its ability to detect intervals of significant workload-related changes in joint moments, even when the peak values shifted temporally across conditions. For the hip abduction moment, SPM revealed a consistent delay in the timing of the peak as workload increased, an effect that scalar analysis failed to capture. This temporal shift may reflect compensatory neuromuscular strategies aimed at stabilizing the pelvis under higher loads.

Such adaptations are biomechanically relevant, as they may influence both performance and injury risk. Delayed or altered moment patterns could indicate increased reliance on specific muscle groups or changes in coordination, potentially leading to overuse injuries such as iliotibial band friction syndrome.

A similar trend can be observed for the peak adduction moment, although no statistically significant differences were found during that phase of the cycle. In the coronal plane, it is evident that an increase in power leads to an increase in the peak external rotation moment (Table 1). The increase in power also appears to delay the timing of the peak external rotation moment; however, this effect did not reach statistical significance (Figure 4). Taken together, these findings enable a non-invasive analysis of hip abductor muscle behaviour, contributing to the detection of potential abnormal neuromuscular patterns.

### 4.2 Knee joint moments

The 0D statistical analysis in the knee provides significant differences in the flexion-extension moment (ROM, Max and Min), the abduction moment (ROM and Min) and in the internal rotation moment (ROM and Max). The difference between the value obtained for P1 and the value obtained for P3 (Table 1) shows that the range of knee flexion torque increases by 49.19 Nm, 78% of the initial value. The peak value of this moment, between the same comparison, increases by 23.06 Nm (75%), and finally, the minimum value becomes more negative, decreasing by 26.12 Nm. Similarly, the range of the knee adductor moment increases by 79% (11.81 Nm), while its minimum value decreases by 11.56 Nm. Finally, the internal rotation moment shows an increase in range of 10 Nm, almost 95%. Its peak value increases by 8.25 Nm, 84% of the initial value. As in the hip, it is of greater interest to analyse the flexion moment minimum (extensor moment maximum) because it occurs during the push phase. However, a similar phenomenon to what occurs in the hip is observed. The increase in workload causes the appearance of a phase of knee flexion moment, abduction and internal rotation moments. According to the paired comparative analysis, these differences can be explained by the increase in power during pedalling, suggesting a different pattern of muscle forces as workload increases. The increased range and magnitude of abduction and internal rotation moments suggest altered recruitment of stabilising muscles such as the vastus medialis and tensor fasciae latae, which may contribute to joint misalignment or increased shear forces (Pouliquen et al., 2021). In similar way to what happened in the hip, the pairwise comparisons showed significance mainly at level P1–P3. Again, this result can be explained by the intravariability increase due to the power intensification.

The SPM shows significance in the sagittal plane but not in the transversal plane. In addition, the SPM shows significance in the pedalling cycle phase where the maximum flexion-extension occur, which take place around the BDC and the differences obtained can be explained by the increase in pedalling power. The superimposition of the temporal evolution of the knee flexion-extension moment shows that the extension maximum shifts to the right as workload increases, the same as occurs with the flexion maximum. This temporal shift may indicate a delayed activation or prolonged engagement of the quadriceps and hamstring muscle groups. Could reflect a compensatory strategy to maintain power output under higher loads, potentially increasing the mechanical demand on the patellofemoral joint (Pouliquen et al., 2021).

Patello-femoral syndrome (“cyclist’s knee”) is the most common cause of pain in cyclists. The pain is caused by increased pressure on the patello-femoral joint. This increase in pressure is caused by the increased in the force exerted (climbing hills, pedalling power, too low cadence). Such increased pressure results in an overload of the articular cartilage which can lead to chondromalacia (Wanich et al., 2007). The use of the SPM allows not only to identify the influence of power on the peak moments but also to evaluate the percentage of the pedalling cycle where these differences are significant, which would allow to evaluate adjustments in the pedalling technique that would reduce the risk of developing this syndrome. Figure 4 illustrates that, during the knee flexion moment, the first and third quarters of the pedalling cycle are the most affected by the increase in pedalling power. This information may be of particular relevance for optimising pedalling technique and for the prevention of overuse injuries. A practical strategy would be to maintain moderate power output while increasing cadence, thereby reducing compressive forces on the patellofemoral joint. These insights could be implemented through real-time feedback systems or training protocols that focus on maintaining joint moments within safe thresholds, particularly in amateur cyclists with less developed neuromuscular control. In the case of the coronal plane, the forces that appear in the knee joint are mainly due to the position of the foot. Foot eversion lowers peak varus and internal axial moments (Cyr and Ascher, 2023; Wanich et al., 2007). This highlights the importance of three-dimensional kinetic analysis of the entire low limb since actions outside the sagittal plane, in this case the foot, influence the possible occurrence of knee complaints. In this study, no influence of increased pedalling power on foot eversion was observed (Figure 5). Future research will aim to investigate the influence of foot eversion on knee joint forces and moments.

### 4.3 Ankle joint moments

The results of the 0D statistical analysis show significant differences in all three planes. In dorsiflexion moment, significance is observed in the range (in all pairwise comparisons) for P1–P2 and P1–P3 comparisons and in the minimum (plantar-flexion maximum) for the P1–P3 and P2–P3 comparisons. In ankle inversion, differences are observed in the range for the three comparisons and in the minimum for P1–P3. Finally, the internal rotation moment shows differences in range and maximum (P1–P3). Based on the values shown in Table 1, for the P1–P3 comparison, the range of the dorsiflexion moment increases by 20.22 Nm, or 118%. The minimum value of this moment decreases by 18.84 Nm. The range of the inversion moment for the same comparison also increases by 14.08 Nm, or 145% in this case. This moment also shows significance in its minimum value, which decreases by 12.93 Nm. Finally, the internal rotation moment of the ankle sees its range and peak value increase by 3.31 Nm (53%) and 2.36 Nm (106%) respectively compared to the value obtained for P1.

SPM provides significant results in all three anatomical planes. Especially in the sagittal and frontal planes, significant differences are found in all three pairwise comparisons during almost the entire push phase. Interestingly, significant results are obtained at the end of the recovery phase in the sagittal and coronal planes. These results suggest that workload has a critical role in the ankle moments, especially in the push phase.

The most common overuse ankle injury in cyclists is Achilles tendinitis. Repeated dorsiflexion during the push phase at high load values can cause this condition. The mechanical factors that contribute to this type of injury are low saddle height, poor pedalling technique and poor foot alignment (Wanich et al., 2007). The results obtained (Table 1; Figure 4) allow for a more precise analysis of the effect of pedalling power on ankle joint moments. Notably, pedalling power appears to influence a substantial portion of the cycle in relation to the dorsiflexion moment. Analysis of the data (Table 1) shows that the peak plantarflexion moment increases in approximately the same proportion as pedalling power, which may provide valuable insights for the design of training programmes and injury prevention strategies. Training programmes that incorporate individualised workload monitoring and progressive overload principles can help athletes improve performance while minimising the risk of overtraining. Additionally, neuromuscular training protocols focusing on core stability, proprioception, and lower limb alignment have been shown to reduce the incidence of injuries such as anterior cruciate ligament tears and patellofemoral pain. Integrating cycling-specific strength training and pedalling technique drills can also enhance power output and efficiency, particularly in competitive cyclists.

Additionally, as the lower body is a closed kinematic chain (Pouliquen et al., 2018; Galindo-Martínez et al., 2021; Buckeridge et al., 2015), what happens in the foot-ankle complex can have consequences in more proximal joints and is briefly discussed below. At the start of the push phase, the ankle is at its maximum degree of dorsiflexion. During the mid-push phase, the calf muscles move the ankle into a plantar-flexion position (Figure 5). Previous research (Bini and Hume, 2020; Holliday et al., 2023) has consistently reported than the soleus and gastrocnemius are active during the middle part of the push phase and the gastrocnemius continues to be active well into the recovery phase. The foot pronates during the push phase due to a dorsiflexion moment equivalent to a force pushing upward on the forefoot. This force causes the midtarsal and subtalar joints to pronate and the medial aspect of the foot to undergo inversion and dorsiflexion (Figure 5). Foot pronation and dorsiflexion of the medial column can reverse the relationship of the forefoot to the leg, which rotates closer to the midline of the body (Sanner and O'Halloran, 2000). This altered alignment may increase the Q-angle and place the knee in a potentially risky position, especially during the extension phase. Although this mechanism could affect the frontal and transverse planes of the knee, no significant effects were observed in our study, possibly due to high inter-individual variability in these planes. However, the sagittal plane effects observed at the knee may be partially explained by this distal-to-proximal transmission of forces within the closed kinematic chain of the lower limb. Consequently, the analysis of the kinematics and dynamics of the foot-ankle complex must have a three-dimensional character in order to explain possible effects on joints such as the knee (Figure 5).

### 4.4 Initial hypothesis evaluation

The results obtained in the statistical analysis show that the moment ranges are much more affected by workload than the maximum and minimum values, which is partly expected, as the ranges depend on both the maximum and minimum values of the joint moments. However, as already discussed, pedalling at different powers can cause changes in the maximum and minimum values and not show significance, but overall, changes in these points can cause the power to statistically affect the range. In the literature consulted, authors have been found who tend to study the maximum and minimum (Fang et al., 2016; Bini and Diefenthaeler, 2010), in other works the ranges are analysed (Pouliquen et al., 2018; Pouliquen et al., 2021), and works have also been found that analyse the average value of the temporal evolution throughout the pedalling cycle (Bini et al., 2010b). As can be seen, there is no unanimity of opinion in the literature on which variables are significant. Despite being a methodology widely used in the literature, statistical analysis using 0D variables has this limitation. Another limitation of this methodology is that when analysing a 0D data, the pedalling cycle is limited to a single variable and the global nature of the pedalling cycle and the factors that are causing it are ignored. For example, when comparing the maximum value of the hip flexion moment for P1 and P3, Figure 4 shows how these significant moments occur in different pedalling cycle phases. Therefore, when performing the 0D statistical analysis of these points, different pedalling cycle phases are being analysed.

Statistical analysis using the SPM methodology allowed to identify the intervals at which significant differences occur. The results of the statistical analysis using 1D variables indicated that workload affected all three joints and all three anatomical planes. However, to the best of knowledge, this is the first study to analyse the effect of pedalling power on joint moments using 1D statistical analysis, so there is no data available in the literature to compare the results obtained.

Once the effect of pedalling power on joint moments has been analysed and compared using 0D and 1D data, the first hypothesis proposed in this work can be evaluated. This stated that the results obtained using 0D variables would be different from the results obtained using the SPM method. This hypothesis is confirmed if the hip results are focused on, where the SPM only provides significant results in the sagittal and frontal plane, whereas the analysis using 0D data provides significant results in all three planes. This result shows that analysing the effect of power using only significant points such as the maximum or range of joint moments can lead to misinterpretation of the results, since, for each power, these points can occur at different times in the cycle (Figure 4). An analysis of the complete cycle comparing each instant of the cycle shows that pedalling power does not have a significant effect on the external rotation moment. Similar conclusions can be reached when analysing the results at the knee outside the sagittal plane.

The results discussed above allow to confirm the second hypothesis proposed in this paper. Workload affects all three joints, whether performing a scalar or parametric statistical analysis. This highlights the importance of analysing the whole of the lower body and not focusing on one joint. Similarly, the results show that pedalling power influences the sagittal, frontal and transverse planes, so that the third hypothesis proposed in this study is also accepted. The confirmation of these two hypotheses highlights the importance of this work, because to the best of knowledge, this is the first article to analyse the effect of pedalling power on the three joints that compose the lower body and on the three anatomical planes. This is important because, as other authors have mentioned in their work (Pouliquen et al., 2018; Galindo-Martínez et al., 2021; Buckeridge et al., 2015), the lower body is a closed kinematic chain and the compensation of the links of this chain to adapt to the new pedalling conditions can affect the rest.

The results shown in this work allow taking decisions for the prevention of the most common overuse injuries in cycling. In addition to quantifying the influence of pedalling power on joint moments, the phases of the cycle where this influence becomes evident can also be identified. Furthermore, the results presented here provide a better understanding of how increased pedalling power modifies joint loading patterns. From a kinematic-chain perspective, the ankle is constrained by the pedal, limiting its degrees of freedom and forcing compensatory adaptations in more proximal joints, particularly the hip. This is consistent with the findings of Pouliquen et al. (2018), who observed that in closed-chain activities, the hip exhibits the greatest asymmetries and adaptations. The observed redistribution of joint moments and their temporal shifts across planes suggest coordinated inter-joint strategies to maintain performance and stability. These findings reinforce the importance of analysing the lower limb as a functional unit. Future work will include a kinetic analysis of the lower limb as a closed-chain system to further explore the mechanisms underlying these adaptations and their implications for injury prevention and performance optimisation.

### 4.5 Limitations

The present study has some limitations that should be taken into account when interpreting the results. Firstly, the sample size, with 10 participants included in the analysis. While this size is consistent with that used in similar studies in the field of cycling biomechanics (Mornieux et al., 2007; Pouliquen et al., 2018; Carpes et al., 2007), a larger number of subjects would have increased statistical robustness, decreasing statistical error and improving the generalisability of the results. Therefore, larger samples should be included in future studies, which would reduce inter-individual variability and increase the statistical power of the analyses.

Other limitation relates to the characteristics of the study sample, which consisted exclusively of males aged between 20 and 35 years, all of whom were amateur cyclists. The inclusion of only male participants was intended to avoid introducing an additional variable, sex, that could potentially affect the outcomes. For instance, this factor could influence the intrinsic Q-factor of the participants (measured from the anterior superior iliac spines) and its relation to the bicycle setup. Similarly, the age range was not extended in order to exclude another variable that might impact the study results. Finally, analysing amateur rather than professional cyclists may limit the extrapolation of the findings to individuals with greater training or competitive experience. Differences in joint moment patterns, mechanical efficiency, and neuromuscular adaptations associated with the level of practice could influence the biomechanical response to variations in pedalling power. However, focusing on amateur cyclists also offers certain advantages, as they represent a substantial proportion of the cycling population. Moreover, unlike professional cyclists, amateurs exhibit greater biomechanical variability, which can facilitate the identification of inefficiencies and injury risks. Studying this group enables personalised recommendations and provides insights applicable to non-competitive real-world contexts. These limitations suggest that future research should investigate whether pedalling power affects males and females differently, include a broader age range to examine how age might influence power effects, and assess the influence of cycling experience by including participants of varying skill levels to better understand how joint moments adapt during pedalling at different power outputs.

Another limitation identified in this study is that only the effect of power on the joint moments of the dominant leg was analysed. Examining both asymmetries in joint moments between legs and the influence of power on the development of such asymmetries is of great interest for improving performance and reducing overload on muscles and joints. For this reason, the authors are currently preparing a separate manuscript addressing these issues.

Finally, an additional limitation concerns the nature of data processing and analysis using SPM, which requires preprocessing steps such as temporal normalisation and signal smoothing (Pataky et al., 2013). While these procedures are necessary to facilitate comparisons between subjects and reduce high-frequency noise, they may influence the shape and magnitude of the significance curves obtained. Furthermore, the calculation of instantaneous joint moments is subject to inherent sources of error related to motion capture, including soft tissue artefacts, variability in marker placement, system calibration and synchronization errors, and assumptions associated with the inverse dynamics model, such as segment inertial properties and joint centre estimations. Although these sources of error are inherent in biomechanical analyses, efforts were made to minimise their impact through standardised protocols, consistent marker placement, use specific marker protocol for cycling, which avoids areas with high soft tissue artefact (Martin-Sosa et al., 2025), functional methods for determining joint centres, and validated instrumentation. Nevertheless, their potential influence on the interpretation of instantaneous joint moment variations should be considered when generalising the findings.

## 5 Conclusion

The aim of this study was to analyse the effect of pedalling power on the hip, knee and ankle joint moments in the 3 anatomical planes using a statistical analysis with 0D data and a statistical analysis with 1D variables. The results obtained are expected to provide information to facilitate the prevention of overuse injuries in the practice of cycling.

Statistical analysis of the effect of workload using 0D variables such as moment ranges and maximum and minimum values showed that pedalling power affected all three joints and all three anatomical planes. Differences were found between the results obtained using 0D data and the SPM. Therefore, the use of both techniques in a complementary way is proposed. The results obtained allow more complete and accurate information to be obtained on the effect of workload on joint moments by combining statistical analyses based on 0D and 1D data. While 0D analysis allows analysis based on indicators such as maximums, minimums or ranges, SPM provides information on the influence of factors such as pedalling power on the temporal evolution pattern of joint moment, even when the maximum or minimum values shifted temporally between different conditions.

These types of results can be of great interest, as seen, when it comes to assessing the potential risks of injury in the three main joints of the lower limb. In the case of the hip, it may be possible to identify the phases of the cycle in which overload occurs due to increased pedalling power, and therefore the increased risk of developing trochanteric bursitis or ITB friction syndrome. For the knee, the use of SPM is of vital importance as it allows the detection of the phases of the pedalling cycle affected by the increase in power and in which there is a greater probability of overload occurring, which could pose a risk of chondromalacia. Furthermore, this study has concluded that it is possible to observe a relationship between increased pedalling power and the maximum moments of hip and knee flexion-extension and ankle dorsiflexion and plantar flexion. As well as the increase in power modifies the temporal pattern and magnitude of the moments in the coronal plane, especially in hip abduction. These findings may allow the establishment of power adjustment criteria to prevent neuromuscular alterations. Similarly, these findings may be of interest for the prevention of injuries related to Achilles tendinitis if the phases of the cycle in which the overload due to increased power becomes critical can be identified.

Pedalling power was found to affect joint moments in the three main joints of the lower body and in the three anatomical planes. These results reinforce the idea proposed by other authors (Pouliquen et al., 2018; Galindo-Martínez et al., 2021; Buckeridge et al., 2015) who suggest that the lower body should be analysed as a whole because it is a closed kinematic chain. Extension of this analysis to joint forces in a similar way to the work of Bini and Hume (2020), including electromyography data, would be of interest. On the other hand, the analysis of other factors such as pedalling cadence or saddle height would also be useful using the techniques employed in this work.

The information provided by the analyses carried out allows for a more precise approach to the prevention of overuse injuries in cycling. According to the literature consulted, several ailments associated with cycling and discussed in this paper are due to actions occurring outside the sagittal plane. The results shown in this work demonstrate that joint moments performed outside this plane can be significantly affected by pedalling power and, therefore, favour the risk of injury.

## Data Availability

The raw data supporting the conclusions of this article will be made available by the authors, without undue reservation.
